# The pyruvate dehydrogenase kinase inhibitor dichloroacetate mitigates alcohol-induced hepatic inflammation and metabolic disturbances in mice

**DOI:** 10.1097/HC9.0000000000000547

**Published:** 2024-11-29

**Authors:** Jianguo Wu, Emily Huang, Megan R. McMullen, Vaibhav Singh, Marko Mrdjen, Annette Bellar, Li Wang, Nicole Welch, Jaividhya Dasarathy, Srinivasan Dasarathy, David Streem, J. Mark Brown, Laura E. Nagy

**Affiliations:** 1 Department of Inflammation and Immunity, Lerner Research Institute, Cleveland Clinic, Cleveland, Ohio, USA; 2 Department of Molecular Medicine, Cleveland Clinic Lerner College of Medicine of Case Western Reserve University, Cleveland, Ohio, USA; 3 Department of Cardiovascular and Metabolic Sciences, Lerner Research Institute, Cleveland Clinic, Cleveland, Ohio, USA; 4 Department of Cancer Biology, Lerner Research Institute, Cleveland Clinic, Cleveland, Ohio, USA; 5 Independent Researcher, Tucson, Arizona, USA; 6 Department of Gastroenterology and Hepatology, Cleveland Clinic, Cleveland, Ohio, USA; 7 Department of Family Medicine, MetroHealth Medical Center, Case Western Reserve University, Cleveland, Ohio, USA; 8 Northern Ohio Alcohol Center, Lerner Research Institute, Cleveland Clinic, Cleveland, Ohio, USA; 9 Department of Psychiatry and Psychology, Cleveland Clinic Lutheran Hospital, Cleveland, Ohio, USA; 10 Center for Microbiome and Human Health, Lerner Research Institute, Cleveland Clinic, Cleveland, Ohio, USA

## Abstract

**Background:**

Dichloroacetate (DCA), a pan-pyruvate dehydrogenase kinase inhibitor, ameliorates multiple pathological conditions and tissue injury and shows strong potential for clinical applications. Here, we investigated the preventive effects of DCA in a murine model of alcohol-associated liver disease.

**Methods:**

C57BL/6J mice were subjected to the acute-on-chronic model of alcohol-associated liver disease and treated with DCA. Livers were assessed in liver histology, biochemistry, and gene expression. Mass spectrometry was used to compare protein expression and metabolite levels.

**Results:**

DCA inhibited hepatic expression of inflammatory genes but did not prevent steatosis and hepatocellular injury in ethanol-fed mice. Consistently, DCA repressed the expression of mRNAs for inflammatory genes in LPS-stimulated murine bone-marrow–derived macrophages and human monocytic THP-1 cells and inhibited both gene expression and protein release of interleukin-1 beta. DCA prevented hepatic accumulation of isovaleric acid in ethanol-fed mice, a short-chain fatty acid primarily produced by gut microbiota. In vitro, isovaleric acid potentiated LPS’s effects, while DCA prevented this proinflammatory action. Ethanol feeding increased the expression of proteins involved in diverse metabolic pathways, including branched-chain amino acid (BCAA) degradation. In ethanol-fed mice, hepatic Fischer’s ratio (the molar ratio of BCAAs to aromatic amino acids Phe and Tyr) and BTR (the molar ratio of BCAAs to Tyr) showed a decrease compared to pair-fed mice; however, this decrease was not observed in DCA-treated ethanol-fed mice. DCA blunted the ethanol-induced increase of BCKDHA, the rate-limiting enzyme in BCAA catabolism, and cytochrome P450 2E1.

**Conclusions:**

Ethanol-induced hepatic inflammatory responses and metabolic disturbances were prevented by DCA in mice, indicating the potential to develop pyruvate dehydrogenase kinase inhibitors as an effective therapy to treat alcohol-associated liver disease.

## Graphical abstract

**Figure:**
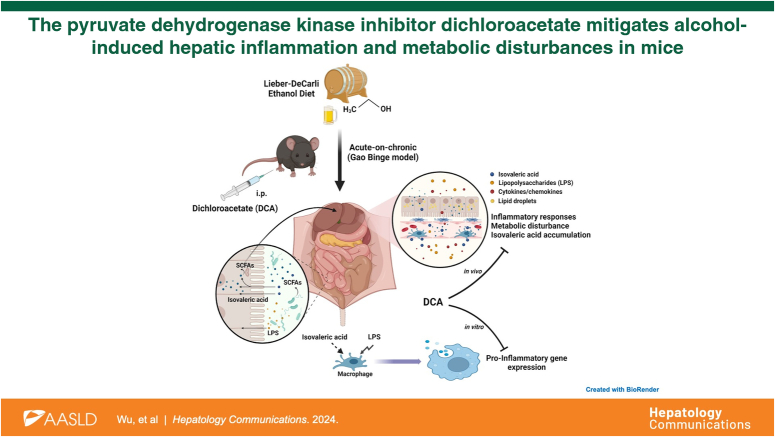

## INTRODUCTION

Alcohol-associated liver disease (ALD), a prominent contributor to liver-related morbidity and mortality, imposes a substantial health burden globally. ALD can progress from steatosis to fibrosis, cirrhosis, and end-stage liver disease or manifest as acute inflammatory syndrome or alcohol-associated hepatitis. ALD stands out as the leading cause of liver transplantation. Multiple interconnected pathophysiological mechanisms contribute to the development and progression of ALD, including inflammatory responses, intestinal barrier leakage and dysbiosis, and metabolic dyshomeostasis.^1^ Indeed, blocking inflammatory signaling represents a potential therapeutic strategy for treating ALD^2^; gut microbial products and meta-organismal metabolic pathways contribute to alcohol-induced liver injury.^3,4^

Lipid homeostasis can be perturbed by alcohol. Fatty acid composition and quantity in the liver, including shifts from saturated fatty acids toward unsaturated fatty acids and elevations of 18-carbon and 22-carbon fatty acids, are implicated in animal models of ALD.^5^ Short-chain fatty acids (SCFAs), ranging from C1 to C6, are primarily generated through intestinal microbial fermentation of dietary fiber. Increasing SCFAs improves gut barrier function,^6^ yet SCFAs can have either proinflammatory or anti-inflammatory activities.^7^ Despite advances in understanding alcohol toxicity, hepatic metabolic disturbances induced by alcohol consumption and the contribution of hepatic SCFAs to liver injury are not completely understood.

Alcohol consumption also disrupts the equilibrium of amino acids. The molar ratio of branched-chain amino acids (BCAAs: leucine, isoleucine, and valine) to aromatic amino acids (tyrosine and phenylalanine; Fischer’s ratio) and the molar ratio of BCAAs to tyrosine (BTR) are valuable diagnostic markers for assessing liver metabolism and hepatic functional reserve.^8^ In addition, both BCAAs and aromatic amino acids can modulate immune cell status and inflammation.^9,10^ The most common abnormality in amino acid profiles in patients with end-stage ALD (and other end-stage liver disease) is a decrease in plasma BCAAs.^11^ Conversely, plasma BCAAs tend to increase in individuals with chronic alcohol consumption.^12^ Despite the limited studies showing that hepatic amino acid profiles and levels are changed in rodent models of ALD,^13,14^ little is known about BCAA metabolism-related gene expression. To better understand the mechanism for BCAA perturbation in ALD, we used ‘omics approaches to assess protein expression and identify changes in metabolite profiles in ethanol-fed mice.

Dichloroacetate (DCA), a structural analog of pyruvate, is the only pan-pyruvate dehydrogenase kinase (PDK) inhibitor that has entered phase II clinical trials for the treatment of congenital lactic acidemia, symptoms of MELAS (mitochondrial myopathy, encephalopathy, lactic acidosis, and stroke-like episodes), and multiple cancers, showing strong potential for clinical applications.^15^ DCA is beneficial in ameliorating pathological conditions and tissue injury, such as lactic acidosis, sepsis, cancers, and inflammation. In cultured hepatocytes, DCA protects cells from ethanol-induced stress by preventing the overwhelming accumulation of reactive oxygen species, lipid, and mitochondrial Ca^2+^ ^16^; however, the effect of DCA on ethanol-induced liver inflammation and metabolic dysfunction has not been determined in vivo.

We hypothesized that DCA protects the liver against the harmful effects of ethanol in mice. Our results demonstrate that DCA mitigates the expression of inflammatory genes, inhibits inflammasome activation, improves metabolite profiles, and reduces hepatic accumulation of isovaleric acid, a gut microbe-derived proinflammatory metabolite. Moreover, in DCA-treated mice, ethanol is no longer able to decrease hepatic Fischer’s ratio and BTR. These findings highlight the beneficial effects of DCA and suggest that PDK inhibition could be employed to develop effective interventions to prevent alcohol consumption-related liver inflammation and metabolic stress.

## METHODS

### Animal models, injection of DCA, and measurement of blood alcohol concentration

Wild-type and *Pdk4* ^*−*/*−*^ mice were handled in accordance with a protocol approved by the Cleveland Clinic Institutional Animal Care and Use Committee. All mice were of C57BL/6J genetic background. Wild-type mice were purchased from Jackson Laboratory (Stock No. 000664). *Pdk4* ^*−*/*−*^ mice, initially generated by Dr Robert A. Harris at Indiana University, were transferred from Dr Adam R. Wende’s laboratory at the University of Alabama at Birmingham. Mice were maintained in clean cages in a 12/12-hour light/dark cycle (lights on from 7 am to 7 pm) with free access to water and chow before experiments. The acute-on-chronic (Gao-binge) ethanol-feeding mouse model has been reported before.^17,18^ In brief, 12-week-old female mice were given a Lieber-DeCarli diet (D710260; Dyets) supplemented with ethanol at 5% (v/v, comprising 28% of total calories) or a pair-fed control diet that isocalorically substituted maltose dextrin for ethanol for 10 days, after acclimation to the pair-fed control diet. On the last day of feeding, mice were gavaged with maltose or ethanol (5 g/kg body weight). Mice were euthanized 6 hours after gavage. DCA (347795-50G; Sigma-Aldrich) or saline (0.9% NaCl) was administered through i.p. injection once daily and right before the binge. Two doses of DCA, 25 mg/kg body weight and 50 mg/kg body weight, were tested. Because the dose of 25 mg/kg body weight was less effective, the results are not shown in the manuscript. Blood was sampled at 2.5 hours in the dark cycle on day 8 of the feeding protocol, and ethanol concentration was measured using an Ethanol L3K kit (#273-30; Sekisui Diagnostics) according to the manufacturer’s instruction.

### Proteomics and targeted metabolomics

The method for preparing peptides for profiling hepatic protein expression in liquid chromatography-tandem mass spectrometry (LC-MS/MS) has been reported previously.^19^ The LC-MS/MS instrument used was the Thermo Scientific Ultimate 3000 nano-flow UHPLC interfaced with an Orbitrap Fusion Lumos Tribrid Mass Spectrometer. Quantification of SCFAs and amino acids in the liver using the targeted metabolomic approach was performed in the Proteomics & Metabolomics Core at Lerner Research Institute, Cleveland Clinic, and detailed in Supplemental Materials and Methods, http://links.lww.com/HC9/B50.

### Untargeted metabolomic analysis of plasma of patients with severe alcohol-associated hepatitis

Patient demographics and clinical data are summarized in Supplemental Table S7, http://links.lww.com/HC9/B49. The recruitment of patients has been described previously.^20^ Deidentified plasma samples from 10 healthy controls and 10 patients with severe alcohol-associated hepatitis (sAH), all recruited at Cleveland Clinic, were obtained from the Northern Ohio Alcohol Center (NCT03224949). Patients were stratified based on disease severity according to the MELD score. sAH is defined as with MELD ≥20. GC-TOF-MS quantification of plasma metabolites was performed by the West Coast Metabolomics Center at the UC Davis Genome Center. The Instruments are Gerstel CIS4 with dual MPS Injector, Agilent 6890 GC, and Pegasus III TOF-MS.

### Statistical analysis

Data are shown as the mean ± SEM. Normal distribution was examined using the Shapiro-Wilk test, and when necessary, data were log-transformed. Statistical comparison was conducted using a Student *t* test or Mann-Whitney test between 2 groups and ANOVA followed by the Tukey test among multiple groups. A difference was considered significant when *p* < 0.05, by GraphPad Prism 9. Supplemental Table S5, S6, http://links.lww.com/HC9/B49.

## RESULTS

### DCA ameliorates hepatic inflammatory responses but not steatosis in ethanol-fed mice

**FIGURE 1:**
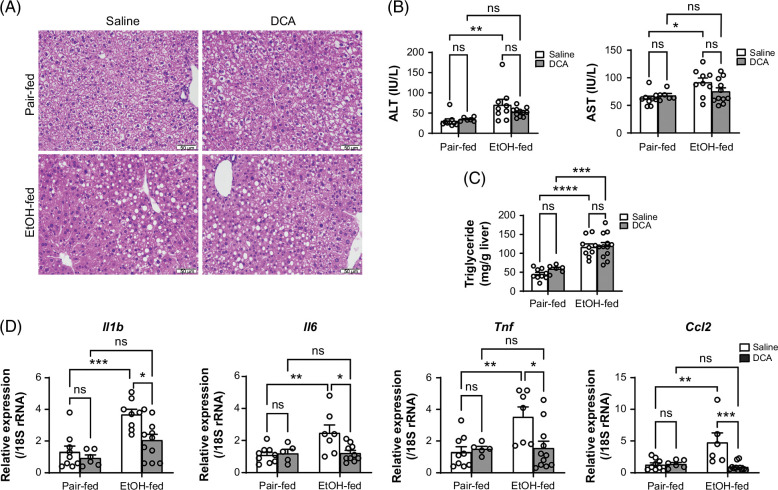
Administration of DCA prevents ethanol-induced inflammatory gene expression rather than hepatocellular injury and steatosis in mice. (A) Representative H&E staining of mouse liver sections. Mice exposed to the acute-on-chronic (Gao-binge) murine model of ALD were injected with saline or DCA. Scale bar, 50 µm. (B–D) Plasma ALT and AST (B), hepatic TGs (C), and qPCR analysis of hepatic gene expression (D) in mice as described in (A). Data are mean ± SEM (n = 5–11 mice/group). *p < 0.05, **p < 0.01, ***p < 0.001, and ****p < 0.0001 by 2-way ANOVA. Abbreviations: ALD, alcohol-associated liver disease; DCA, dichloroacetate; H&E, hematoxylin and eosin; qPCR, quantitative polymerase chain reaction; TG, triglycerides.

To assess the potential of DCA to alleviate ethanol-induced liver injury, we administered DCA to mice subjected to the acute-on-chronic model in which hepatic expression of PDK3 and PDK4 proteins were increased by ethanol (Supplemental Figure S1A, http://links.lww.com/HC9/B50). Compared to pair-fed control mice, ethanol-fed mice exhibited increased plasma levels of ALT and AST, elevated liver triglycerides, histologic evidence of hepatic macrovesicular steatosis, and upregulation of mRNAs for inflammatory mediators including *Il1b*, *Il6*, *Tnf*, and *Ccl2* (Figures 1A–D). Treatment with DCA effectively prevented the ethanol-induced upregulation of mRNAs for *Il1b*, *Il6*, *Tnf*, and *Ccl2* (Figure 1D) but did not prevent other manifestations of ethanol-induced liver toxicity (Figures 1A–C). In addition, DCA inhibited the ethanol-induced expression of pro-IL1B (interleukin-1 beta) protein (Supplemental Figure S1B, http://links.lww.com/HC9/B50). The final body weight was decreased by ethanol in DCA-treated but not in saline-treated mice (Supplemental Figure S1C, http://links.lww.com/HC9/B50), possibly due to DCA’s ability to increase systemic energy expenditure.^21^ Interestingly, although DCA prevented the increase in liver/body weight ratio observed in ethanol-fed mice, it induced hepatomegaly in pair-fed mice (Supplemental Figure S1D, http://links.lww.com/HC9/B50). The ethanol concentration in the blood of ethanol-fed mice was unaffected by DCA on day 8 of the feeding trial (Supplemental Figure S1E, http://links.lww.com/HC9/B50). Collectively, these findings demonstrate the impact of DCA on ethanol-induced liver injury, highlighting its efficacy in inhibiting the expression of inflammatory genes.

### DCA suppresses IL1B expression and release

**FIGURE 2:**
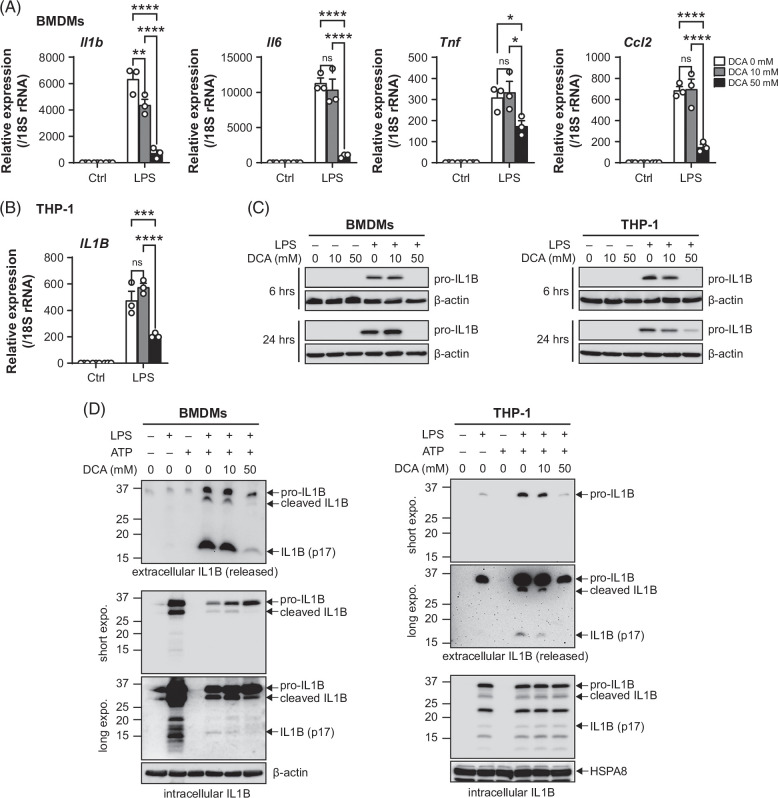
DCA blunts LPS’s effects and inhibits IL1B gene expression and protein release. (A, B) qPCR of gene expression in BMDMs (A) and THP-1 cells (B) either untreated (medium control, ctrl) or treated with LPS (10 ng/mL, 6 h) with or without the presence of DCA. Data are mean ± SEM of 3 independent assays; *p < 0.05, **p < 0.01, ***p < 0.001, ****p < 0.0001; ns, not significant; 2-way ANOVA. (C) Western blot of the indicated proteins in whole-cell lysates from BMDMs or THP-1 cells either untreated (medium control, −) or treated with LPS (10 ng/mL, +) with or without the presence of DCA for the indicated time. (D) Western blot analysis of IL1B levels in IL1B release assays. BMDMs (left) or THP-1 cells (right) were primed with LPS (300 ng/mL, 3 h) and activated with ATP (5 mM, 1 h), in the presence of different concentrations of DCA. (C, D) Data are representative of 3 independent experiments. Abbreviations: BMDM, bone-marrow–derived macrophage; DCA, dichloroacetate; IL1B, interleukin-1 beta; LPS, lipopolysaccharide.

Immune cells from patients with ALD exhibit heightened sensitivity to inflammatory signals, responding with exacerbated inflammatory reactions.^22^ We investigated whether DCA inhibits the expression of inflammatory mediators in cultured cells. DCA, with mM-grade apparent inhibitory constant (Ki) values for PDKs, has been tested in a wide range of concentrations (up to 100 mM) in cell studies.^23,24,25^ We tested a lower concentration (10 mM) to determine its effectiveness and a higher concentration (50 mM) to elicit significant cellular responses. In cultured LPS-stimulated bone-marrow–derived macrophages (BMDMs), DCA inhibited the expression of mRNAs for *Il1b*, *Il6*, *Tnf*, and *Ccl2* (Figure 2A). The inhibition of expression of mRNAs for IL1B was further confirmed in human monocytic THP-1 cells (Figure 2B). IL1B is a potent proinflammatory cytokine and is increased in patients with ALD.^26^ IL1B is produced from the cleavage of pro-IL1B by caspase-1, which is activated by inflammasomes. Expression of pro-IL1B protein was completely abolished by treatment with 50 mM of DCA for 6 hours or 24 hours in both BMDMs and THP-1 cells (Figure 2C). In IL1B release assays, inhibition of extracellular levels of mature IL1B (p17), cleaved IL1B, and pro-IL1B in BMDMs was apparent only at 50 mM of DCA, which coincided with the accumulation of intracellular pro-IL1B and a decrease of intracellular mature IL1B (p17) and cleaved IL1B (Figure 2D, left). The extracellular mature IL1B (p17), cleaved IL1B, and pro-IL1B released from THP-1 cells were all dose-dependently decreased by DCA, while their intracellular levels were not affected (Figure 2D, right). IL1B release was unaffected in *Pdk4* ^*−*/*−*^ BMDMs compared to wild-type BMDMs, suggesting that IL1B release is independent of PDK4 (Supplemental Figure S2, http://links.lww.com/HC9/B50). Taken together, these results suggest that DCA exhibits anti-inflammatory properties in vitro by reducing cytokine production.

### DCA impacts hepatic concentrations of SCFAs and mitigates hepatic accumulation of isovaleric acid in ethanol-fed mice

**FIGURE 3:**
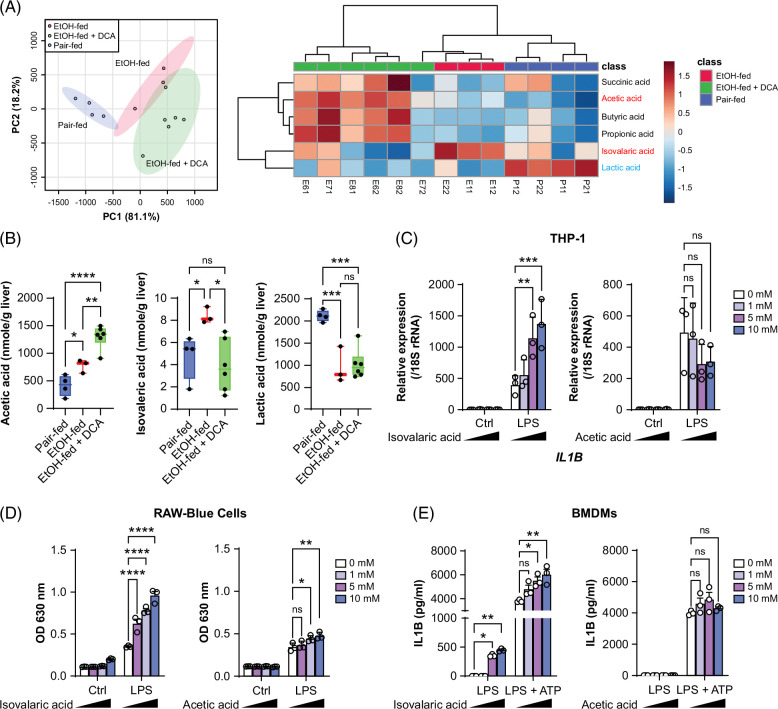
Ethanol feeding in mice leads to hepatic accumulation of SCFAs, including isovaleric acid, which potentially exhibits a proinflammatory property. (A) PCA plot (left) and heatmap (right) showing distinct SCFA composition and changes in SCFA concentrations, quantified by GC-MS/MS, in the indicated groups of mouse livers exposed to the acute-on-chronic model. n = 3–6/group. (B) Concentrations of indicated SCFAs as described in (A). *p < 0.05, **p < 0.01, ****p < 0.0001; ns, not significant; one-way ANOVA. (C) qPCR of *IL1B* mRNA expression in THP-1 cells pretreated with indicated concentrations of isovaleric acid or acetic acid for 3 hours, followed by challenge with or without (control, ctrl) addition of LPS (10 ng/mL) for another 3 hours. (D) RAW-Blue cell reporter assay. 2 × 10^4^ RAW-Blue cells were seeded into each well of 96-well plates, treated with indicated concentrations of isovaleric acid or acetic acid for 3 hours, then stimulated with or without (control, ctrl) addition of LPS (10 ng/mL) for 21 hours. Cell culture medium was collected and assayed. (E) ELISA of IL1B release assays. 5 × 10^4^ BMDMs were seeded into the individual wells of a 96-well plate, treated with the indicated concentrations of isovaleric acid or acetic acid for 3 hours, primed by the addition of LPS (300 ng/mL) for another 3 hours, then stimulated with addition of ATP (5 mM) for 3 hours. The cell culture medium was collected, spun to remove debris, and assayed for IL1B. (C–E) Data are mean ± SEM of 3 independent assays; *p < 0.05, **p < 0.01, ***p < 0.001, ****p < 0.0001; ns, not significant; 2-way ANOVA. Abbreviations: BMDM, bone marrow-derived macrophage; GC-MS/MS, gas chromatography-tandem mass spectrometry; IL1B, interleukin-1 beta; LPS, lipopolysaccharide; PCA, principal component analysis; qPCR, quantitative polymerase chain reaction; SCFA, short-chain fatty acid.

In addition to inducing inflammation, alcohol consumption disturbs lipid homeostasis. We determined the concentrations of 6 SCFAs in the liver using gas chromatography-tandem mass spectrometry. Ethanol feeding increased hepatic acetic acid and isovaleric acid concentrations but decreased lactic acid concentrations in mice. However, ethanol did not affect succinic acid, butyric acid, and propionic acid concentrations. In ethanol-fed mice, DCA treatment increased succinic acid, acetic acid, butyric acid, and propionic acid concentrations and restored isovaleric acid concentrations without affecting lactic acid concentrations (Figures 3A, B, and Supplemental Table S1, http://links.lww.com/HC9/B49). SCFAs, either obtained from diets or derived from gut microbiota, can modulate metabolism, cell signaling, inflammation, etc.^27,28,29^. SCFAs exhibit gradient and variable concentrations within the gut lumen, ranging from 20 to 140 mM, with a small proportion reaching the liver.^27^ Based on a previously reported range of the working concentrations of SCFAs, we tested the immunomodulatory activity of isovaleric acid and acetic acid at 1–10 mM in cell cultures.^27^ In contrast to acetic acid, isovaleric acid dose-dependently potentiated LPS-induced expression of IL1B mRNA (Figure 3C). In the reporter RAW-Blue cells, which monitor the NF-κB and AP-1 responses upon pattern recognition receptor stimulation, isovaleric acid increased LPS-stimulated reporter activity more robustly than acetic acid (Figure 3D). Moreover, isovaleric acid, but not acetic acid, increased IL1B release in LPS-primed and ATP-stimulated BMDMs. Isovaleric acid was sufficient to release small amounts of IL1B in LPS-primed BMDMs, albeit much less potent than ATP (Figure 3E). Taken together, these findings in cultured immune cells suggest that DCA’s anti-inflammatory effects on ethanol-fed mice are associated, at least in part, with its ability of altering hepatic SCFA concentrations.

### DCA inhibits the IL1B-stimulating action of isovaleric acid in cultured immune cells

**FIGURE 4:**
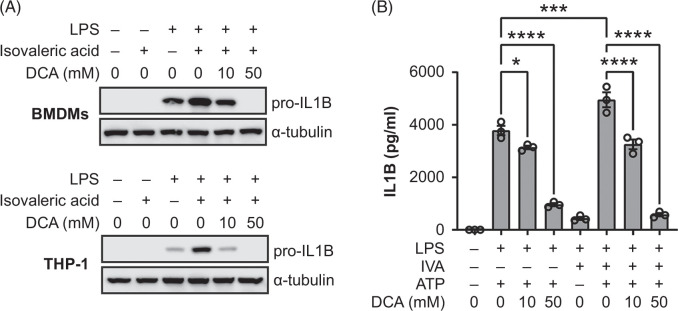
DCA impairs the synergistic effect of isovaleric acid on LPS. (A) BMDMs and THP-1 cells were either left untreated (−) or treated with LPS (10 ng/mL), isovaleric acid (5 mM), and/or DCA (10 mM or 50 mM) for 24 hours, followed by collection for preparation of whole-cell lysates and western blot. (B) ELISA of IL1B release assays. 5 × 10^4^ BMDMs were seeded into the individual wells of a 96-well plate, primed by LPS (300 ng/mL) with or without the presence of isovaleric acid (5 mM), and then activated with the addition of ATP (5 mM) with or without the combination of DCA (10 mM or 50 mM) for 3 hours. Cell culture medium was collected, spun to remove debris, and assayed for IL1B. *p < 0.05, ***p < 0.001, ****p < 0.0001; one-way ANOVA; n = 3. Abbreviations: BMDM, bone-marrow–derived macrophage; DCA, dichloroacetate; IL1B, interleukin-1 beta; LPS, lipopolysaccharide.

To further understand the ability of DCA to prevent the proinflammatory effect of isovaleric acid, BMDMs or THP-1 cells were costimulated with isovaleric acid and LPS and treated with DCA. While isovaleric acid alone did not induce expression of pro-IL1B protein, it synergized with LPS to do so; however, this synergistic effect was remarkably inhibited by DCA (Figure 4A). DCA inhibited IL1B release in LPS-primed and ATP-stimulated BMDMs (Figure 4B), which is consistent with the previous results of western blots (Figure 3E). Notably, IL1B release from LPS-primed and ATP-stimulated BMDMs was more pronounced in the presence of isovaleric acid, an effect dose-dependently compromised by DCA (Figure 4B). Taken together, these results suggest that DCA antagonizes the IL1B-stimulating effect of isovaleric acid.

### Ethanol upregulates the expression of proteins involved in diverse metabolic pathways and DCA reduces expression of metabolic enzymes in ethanol-fed mice

**FIGURE 5:**
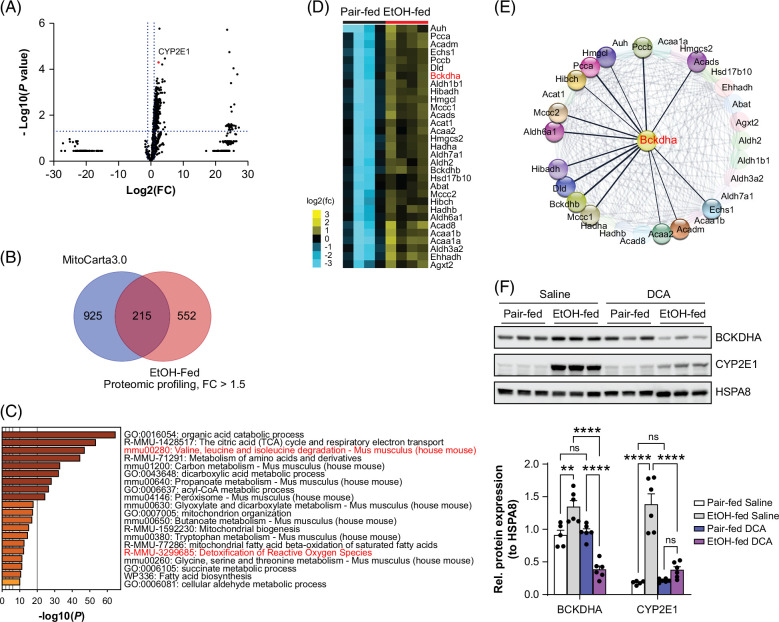
Ethanol feeding increases the expression of multiple metabolic proteins, while DCA reduces ethanol-induced expression of BCKDHA and CYP2E1 in mice. (A) The volcano plot showing LC-MS/MS-based relative quantification of the expression of proteins extracted from the livers of pair-fed and ethanol-fed mice in the acute-on-chronic model. The comparison was based on LFQ intensity; n = 4/group, 2-tailed *t* test. CYP2E1, a known indicator of ethanol uptake, was indicated in the plot. (B) The Venn diagram demonstrating that a large portion of proteins increased by ethanol was mitochondrial proteins. The upregulated proteins identified in (A) and with a fold change of more than 1.5 were matched to the proteins collected in Mitocarta 3.0. (C) The mitochondrial proteins increased by ethanol, identified in (B), were analyzed for gene annotation and ontology using Metascape, and the top 20 involved pathways/biological processes were presented in the bar graph. (D) The heatmap showing the increase of mitochondrial proteins involved in the process of “mmu00280: BCAA degradation” identified in (C) in the mouse livers as described in (A). (E) STRING analysis of protein physical- and functional-interaction network of BCKDHA with the increased proteins from (D). (F) Western blot (top) and quantification using ImageJ (bottom) of the indicated proteins in the livers of mice exposed to the acute-on-chronic model with or without DCA treatment (50 mg/kg body weight). Representative blots were shown, n = 6–11; data are mean ± SEM; **p < 0.01, ****p < 0.0001; ns, not significant; 2-way ANOVA. Abbreviations: BCAA, branched-chain amino acid; BCKDHA, branched-chain keto acid dehydrogenase E1 subunit alpha; CYP2E1, cytochrome P450 2E1; DCA, dichloroacetate; LC-MS/MS, liquid chromatography-tandem mass spectrometry; LFQ, label-free quantitation.

The changes in hepatic SCFAs, particularly isovaleric acid, prompted us to assess ethanol-induced changes in metabolic pathways and related gene expression in the liver. Isovaleric acid is mainly produced by bacterial fermentation of leucine in the colon but relies on the metabolic pathway of BCAAs for catabolism. Proteomic profiling of hepatic proteins using mass spectrometry–based relative quantitative methods revealed that acute-on-chronic ethanol administration in mice remarkably upregulated rather than downregulated protein expression, with a total of 2676 hits and 767 significantly upregulated proteins (fold change >1.5) (Figure 5A, Supplemental Figure S3, http://links.lww.com/HC9/B50, and Supplemental Table S2, http://links.lww.com/HC9/B49). Two hundred fifteen out of the 767 upregulated proteins were identified to be located within the mitochondria (Figure 5B). Gene annotation and enrichment analysis of these 215 upregulated mitochondrial proteins using the web tool Metascape revealed their involvement in multiple metabolic processes, including the metabolism of organic acids, amino acids, carbons, fatty acids, and aldehydes. In addition, they were implicated in the citric acid (TCA) cycle, respiratory electron transport, and mitochondrion organization and biogenesis (Figure 5C). Among these upregulated mitochondrial proteins, 31, including BCDKHA (branched-chain keto acid dehydrogenase E1 subunit alpha), the rate-limiting enzyme for BCAA catabolism, were involved in BCAA degradation, which was among the top enriched pathways (Figures 5C and D). STRING network analysis identified that BCDKHA either physically or functionally interacted with other upregulated enzymes involved in BCAA metabolism within the mitochondria (Figure 5E). Western blot confirmed that ethanol feeding increased hepatic expression of BCDKHA (Figure 5F). Administration of DCA inhibited expression of BCDKHA protein in ethanol-fed mouse livers. Notably, DCA also reduced ethanol-induced expression of cytochrome P450 2E1 (CYP2E1) protein, which oxidizes alcohol and leads to oxidative stress (Figures 5A and F). Collectively, these findings suggest that DCA can attenuate the metabolic disruptions induced by ethanol, partly by modulating BCAA metabolism and CYP2E1 expression.

### DCA improves BCAA balance in ethanol-fed mice

**FIGURE 6:**
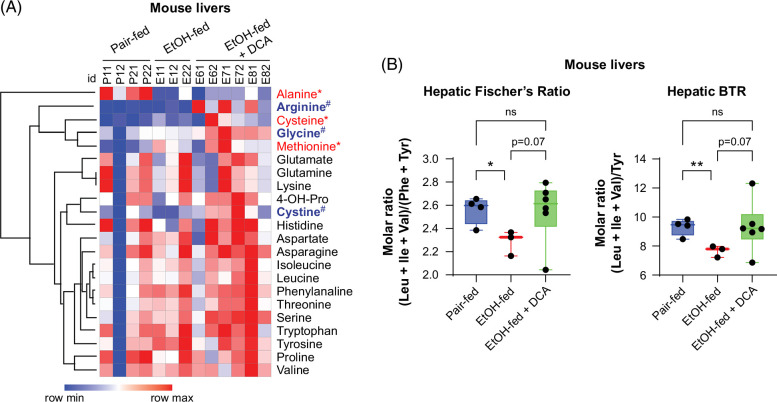
Hepatic amino acid changes in ethanol-fed mice with or without administration of DCA. (A) The heatmap displaying amino acid levels in the livers of indicated groups of mice exposed to the acute-on-chronic ethanol feeding model measured using LC-MS/MS. Amino acids labeled in red indicate differences between ethanol-fed and pair-fed mice (*p < 0.05), while those in blue denote differences between ethanol-fed + DCA and ethanol-fed mice (^#^ *p* < 0.05), analyzed using one-way ANOVA. DCA, 50 mg/kg body weight. (B) Hepatic Fischer’s ratio and BTR in mice as described in (A). *p < 0.05, **p < 0.01; ns, not significant; one-way ANOVA. Abbreviations: BTR, molar ratio of BCAAs to tyrosine; DCA, dichloroacetate; LC-MS/MS, liquid chromatography-tandem mass spectrometry.

To further understand the impact of DCA on metabolism in ethanol-fed mouse livers, we analyzed hepatic levels of amino acids using LC-MS/MS. The livers from ethanol-fed mice exhibited a decrease in alanine but an increase in cysteine and methionine compared to those from pair-fed mice. In ethanol-fed mice, administration of DCA resulted in the elevation of arginine, glycine, and cystine in the liver, suggesting that DCA might skew metabolic processes involving these amino acids (Figure 6A and Supplemental Table S3, http://links.lww.com/HC9/B49). The level of arginine was positively correlated with that of glycine; methionine can be converted to cysteine; indeed, its level was positively correlated with that of cysteine (Supplemental Figure S3A, http://links.lww.com/HC9/B50 and Supplemental Table S4, http://links.lww.com/HC9/B49). The levels of BCAAs and aromatic amino acids were not individually altered in the livers of ethanol-fed mice (Supplemental Figure S3B, http://links.lww.com/HC9/B50). However, the administration of ethanol decreased the hepatic Fischer’s ratio and BTR, while DCA-treated ethanol-fed mice exhibited no differences compared to pair-fed mice (Figure 6B). This finding is consistent with the results that ethanol facilitated the pathway of BCAA degradation (Figure 5C), and DCA inhibited expression of the BCAA catabolic enzyme BCKDHA (Figure 5F).

### Plasma BCAA levels are decreased in patients with sAH

**FIGURE 7:**
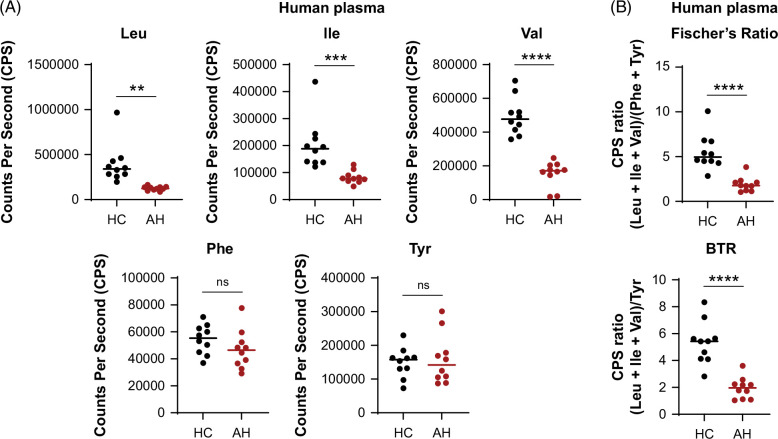
Plasma BCAA and aromatic amino acid alternations in patients with sAH. (A) Plasma from HCs or patients with sAH (AH) was analyzed using GC-TOF-MS to determine the levels of BCAAs, phenylalanine, and tyrosine. CPS, corresponding to the number of ions striking the detector every second, represents the relative abundance of amino acids in the sample. (B) Fischer’s ratio and BTR were calculated based on CPS values from (A). n = 10; data are mean ± SEM; **p < 0.01, ***p < 0.001, ****p < 0.0001; ns, not significant; unpaired *t* test. Abbreviations: BCAA, branched-chain amino acid; BTR, molar ratio of BCAAs to tyrosine; CPS, counts per second; GC-TOF-MS, Gas Chromatography Time-of-Flight Mass Spectrometry; HC, healthy controls; sAH, severe alcohol-associated hepatitis.

Untargeted metabolomic quantification of plasma from patients with sAH revealed a decrease in the levels of BCAAs but not aromatic amino acids (Figure 7A). This decrease was consistent with the observed reduction in Fischer’s ratio and BTR, as previously reported (Figure 7B).^30^

## DISCUSSION

Ethanol-mediated hepatocellular injury, inflammatory immune responses to injury, and intestinal barrier leakage and dysbiosis are the 3 dominant concepts underlying the pathogenesis of ALD.^1^ In this study, we demonstrate that the pan-PDK inhibitor DCA reduces ethanol-induced hepatic inflammatory responses in the acute-on-chronic murine model of ALD. This reduction is mechanistically related to changes in the metabolic profile of SCFAs and amino acids and a decrease in ethanol-induced accumulation of the gut microbiome-derived metabolite isovaleric acid in the liver.

The anti-inflammatory effect of DCA in cultured cells and ethanol-fed mice, reported here, aligns with previous studies in various inflammatory conditions, such as cecal ligation and puncture-induced sepsis.^31^ Specifically, we observed that DCA inhibits both the expression and release of IL1B (Figure 2). DCA can skew pyruvate metabolism from lactate production in the cytoplasm to oxidative production of acetyl-CoA in the mitochondria. Arginine enhances mitochondrial oxidative phosphorylation while dampening glycolysis.^32^ In this regard, increased arginine in DCA-treated ethanol-fed mouse livers is in agreement with DCA’s effect (Figure 6A). Interestingly, despite its role as an efficient inhibitor of aerobic glycolysis, the anti-inflammatory effect of DCA in ethanol-fed mouse livers does not seem to be related to its impact on aerobic glycolysis, as evidenced by unaffected lactate levels (Figure 3B). Instead, we observed an increase in succinic acid levels with DCA treatment, suggesting expedited oxidative phosphorylation and potentially higher levels of TCA intermediates. Increased oxidative phosphorylation generally favors an anti-inflammatory phenotype in activated M2 macrophages and regulatory T cells, which may partially explain the anti-inflammatory effect of DCA in ethanol-fed mouse livers.^33^

Isovaleric acid, also known as β-methylbutyric acid or 3-methylbutanoic acid, is a catabolic product of the essential amino acid leucine. It can be produced by several gut bacterial flora, primarily those involved in protein and amino acid fermentation, including *Clostridium sporogenes*, *Peptostreptococcus*, *Lactobacilli*, *Lachnospiraceae*, etc.^29,34^ Patients with different stages of ALD exhibit significant enteric dysbiosis. *Lactobacilli* and *Lachnospiraceae* are reduced in the gut of patients with ALD.^35,36^ There is little direct evidence showing how other enteric isovaleric acid-producing bacteria are changed in patients with ALD. A study shows that lower levels of isovalerate in fecal SCFAs are correlated with higher alcohol consumption in patients with alcohol use disorder.^37^ Despite findings that a waning production of certain SCFAs is postulated to increase gut permeability and contribute to hepatic inflammation in ALD, the mechanisms contributing to hepatic accumulation of isovaleric acid remain elusive, and the contribution of gut microbiota-derived isovaleric acid to liver injury needs further investigation. Gut microbiome-derived isovaleric acid exhibits IgA-modulatory activity in mice and regulates macrophage polarization.^29,38^ Our study reveals, for the first time, that alcohol consumption can lead to hepatic accumulation of isovaleric acid in mice, highlighting the active involvement of the gut-liver axis in the pathogenesis of ALD. Moreover, we find that isovaleric acid facilitates LPS-induced IL1B expression and release (Figure 3). Isovaleric acidemia is an autosomal recessive inborn error of leucine metabolism arising from a defect in the mitochondrial enzyme isovaleryl-CoA dehydrogenase, resulting in the accumulation of derivatives of isovaleryl-CoA, such as glycine conjugation and isovaleric acid.^39^ Whether DCA regulates isovaleryl-CoA dehydrogenase activity, the catabolism of isovaleryl-CoA, and the gut microbiome composition in ethanol-fed mice remains unknown. However, if so, each of these potential mechanisms might contribute to the hepatic accumulation of isovaleric acid. Unlike other reported anti-inflammatory SCFAs, the specific molecular mechanisms by which isovaleric acid exerts its proinflammatory activity remain unclear. Further investigation is needed to elucidate its binding receptors, transporters, and signaling pathways, which will shed light on its role in inflammation.

Our proteomic data demonstrate that proteins involved in diverse metabolic processes are increased in ethanol-fed mouse livers (Figures 5A–C). CYP2E1 plays a major role in blunting ethanol-induced oxidative stress.^40^ Decreased hepatic expression of CYP2E1 in DCA-treated, ethanol-fed mice suggests that DCA mitigates ethanol-induced oxidative stress (Figure 5F), consistent with previous findings on its neuroprotection effects.^41^ Despite the increase of proteins involved in BCAA degradation, hepatic BCAAs and aromatic amino acids are not individually changed in ethanol-fed mice, which is possibly due to rapid replenishment and yet to be manifested in the development of ALD. Ethanol feeding enhances mTOR signaling in mice.^42^ We did not find that DCA remarkably affected the activity of mTORC1 signaling in vivo (data not shown). The impact of DCA on cell signaling pathways needs further investigation.

Steatosis and inflammation are interconnected processes in the development of ALD. In most models, agents that protect against inflammation also tend to decrease steatosis. Why does DCA not mitigate hepatic steatosis in the acute-on-chronic ethanol mouse model? PDKs are central to carbohydrate metabolism but are differentially expressed in various cells and tissues to coordinate nutrient utilization. The development of hepatic steatosis involves multiple steps of lipid metabolism, including fatty acid uptake, de novo lipogenesis, lipid droplet formation, fatty acid oxidation, and lipoprotein secretion, all of which could be affected by systemic inhibition of PDKs by DCA. Factors such as the dosing of DCA, the length of ethanol feeding, and the timing of experimental endpoints also influence the final severity of hepatic steatosis. DCA ameliorates sepsis-induced steatosis.^31^ Inhibition of PDK2 prevents hepatic steatosis induced by a high-fat diet in mice.^43^ Genetic depletion of PDK4 ameliorates ethanol-induced hepatic steatosis but enhances resection-induced transient regeneration–associated steatosis in mice.^16,44^ These findings suggest that while PDKs regulate hepatic lipid accumulation, targeting them in the context of different pathophysiological processes requires further investigation.

It is important to note that the primary metabolism of DCA involves its conversion to glyoxylate, catalyzed by GSTZ1 (glutathione transferase zeta 1) (also known as maleylacetoacetate isomerase, MAAI). GSTZ1 can be irreversibly inactivated by DCA, leading to an extended half-life of DCA.^45^ This “off-target” effect of DCA should be considered when interpreting its action since GSTZ1, abundant in the liver, catalyzes the glutathione-dependent isomerization of maleylacetoacetate to fumarylacetoacetate, the second-to-last step in the phenylalanine and tyrosine degradation pathway.^46^ This impact might partially contribute to the potential effect of DCA on Fischer’s ratio and BTR (Figure 6B). Glyoxylate can be further broken down into glycine, pyruvate, or oxalate, which might explain the increased hepatic glycine in DCA-administered ethanol-fed mice (Figure 6A).^47^

Despite the beneficial effects, DCA is well-known for its adverse effect of potentially causing reversible peripheral neuropathy with chronic administration.^48^ That DCA induces hepatomegaly has been reported before and is likely associated with glycogen accumulation in hepatocytes.^49^ The US Environmental Protection Agency has reported that DCA can increase liver weight (both absolute and relative) in rodents, attributed to the enlargement of liver cells (Report Number: EPA 815-B-98-010; https://nepis.epa.gov/Exe/ZyPURL.cgi?Dockey=91019ECU.txt). No epidemiologic data suggest DCA is carcinogenic in humans. Further investigation of other PDK inhibitors may help elucidate whether this adverse effect is related to PDK inhibition.

In summary, our work demonstrates that administration of DCA exhibits certain beneficial effects in protecting the liver against ethanol-induced injury by mitigating inflammatory responses, altering the expression of liver metabolic enzymes, and shifting metabolite profiles. Our findings shed light on the pathogenesis of ALD and advance our understanding of ethanol’s effect on inflammation, metabolic homeostasis, and gut-liver crosstalk. Our study highlights the potential of harnessing metabolic enzymes as promising therapeutic approaches to combat ALD.

## Supplementary Material
