# Supplementary material for: The pyruvate dehydrogenase kinase inhibitor dichloroacetate mitigates alcohol-induced hepatic inflammation and metabolic disturbances in mice
Source: Hepatol Commun. 2024 Nov 29;8(12):e0547. doi: 10.1097/HC9.0000000000000547 (PMC11608733; doi:10.1097/HC9.0000000000000547)
Supplement: Supplementary file 2 [file hc9-8-e0547-s002.docx]

The pyruvate dehydrogenase kinase inhibitor dichloroacetate mitigates alcohol-induced hepatic inflammation and metabolic disturbances in mice

Authors: Jianguo Wu, Emily Huang, Megan R. McMullen, Vaibhav Singh, Marko Mrdjen, Annette Bellar, Li Wang, Nicole Welch, Jaividhya Dasarathy, Srinivasan Dasarathy, David Streem, J. Mark Brown, Laura E. Nagy

Supporting Information 1

Contents

[**Supporting Materials and Methods** 2](#_Toc167884941)

[**Supporting Fig. 1** 3](#_Toc167884942)

[**Supporting Fig. 2** 4](#_Toc167884943)

[**Supporting Fig. 3** 5](#_Toc167884944)

[**Supporting Fig. 4** 6](#_Toc167884945)

# **Supporting Materials and Methods**

***Histology and liver and serum biochemistry assays***

Formalin-fixed tissues were paraffin-embedded, sectioned, and stained with hematoxylin and eosin (H&E) for histological analysis. Plasma samples were assayed for alanine aminotransferase (ALT) and aspartate aminotransferase (AST) levels using commercial kits (#318-30 and #319-30; Sekisui Diagnostics). Total liver triglyceride (TG) was quantified using a Triglyceride (GPO) (Liquid) Reagent Set (T7532500, Pointe Scientific, Canton, MI). All procedures were performed following the manufacturer’s instructions.

***Tissue culture, differentiation of BMDMs, and reagents***

The human monocytic cell line THP-1 was originally bought from ATCC and was tested for mycoplasma contamination. Mouse bone-marrow-derived macrophages (BMDMs) were prepared by differentiating isolated bone-marrow cells with 20 ng/ml Recombinant Murine M-CSF (#315-02; PeproTech, Cranbury, NJ) for 10 days. The cells were cultured in HEPES buffered DMEM/F-12 supplemented with 10% fetal bovine serum (FBS), 2 mM glutamine, and 100 U/ml penicillin-streptomycin at 37°C in a humidified incubator with 5% CO_2_. Isovaleric acid was purchased from MilliporeSigma (#129542; Burlington, MA).

***Reverse transcription-quantitative PCR (RT-qPCR) and Western blot***

Total RNA was isolated with Direct-zol RNA Microprep or Miniprep Plus kit (R2062 or R2072; Zymo Research, Irvine, CA) and reverse transcribed into cDNA using the SuperScript™ IV VILO™ Master Mix (#11756500; Thermo Fisher Scientific, Waltham, MA). Real-time PCR was performed in the QuantStudio^TM^ 5 with default cycles. The data were analyzed using the Comparative CT method and normalized to 18S rRNA. Primer sequences are listed in Supporting Table 5. Detailed methods for cell lysate preparation and western blot have been described previously [^1^](#_ENREF_1)^,^[^2^](#_ENREF_2). The antibodies used are listed in Supporting Table 6.

***IL1B release assay and ELISA***

BMDM or THP-1 cells were primed with LPS (300 ng/ml, 3 h) and IL1B release was triggered with ATP (5 mM, 1 h or 3 h). After stimulation, the cell culture medium containing released IL1B was collected (THP-1 cells requires centrifugation, 300 × g for 5 min at 4 °C) and further centrifuged (1400 × g for 2 min at 4 °C) to remove debris and/or dead/unhealthy cells, generating a cell-free preparation. IL1B concentration in the medium was assayed using an ELISA MAX™ Standard Set Mouse IL-1β kit (#432601; BioLegend) according to the manufacturer’s instruction. Alternatively, the medium and the cell pellets were analyzed by western blot.

***RAW-Blue cell reporter assay***

RAW-Blue™ cells (#raw-sp; Invivogen, San Diego, CA) are designed to monitor the NF-κB and AP-1 responses upon pattern recognition receptor stimulation by measuring the activity of secreted embryonic alkaline phosphatase. Cells were seeded into 96-well plates at a density of 2 x 10^4^/well, pre-treated with different concentrations of isovaleric acid or acetic acid for 3 h, and then stimulated with or without (medium control) the addition of LPS (10 ng/ml) for 21 h. Afterward, the cell culture medium was collected and spun to remove debris. A 20 µl of aliquot per well was assayed according to the manufacturer’s instruction, with an incubation time of 2.5 h at 37°C and a read at OD630.

**Targeted metabolomics**

SCFAs were determined using TSQ 8000 Evo Triple Quadrupole GC-MS/MS (ThermoFisher). In brief, liver pieces were weighed, homogenized with 0.5 ml of 0.005 M NaOH with agitation for 20 min at 4°C. The supernatants were collected after centrifugation. A 20 μl aliquot of supernatant was mixed with 50 μL 2‐Butanol/Pyridine (3:2) containing the heavy-labeled internal standards. The carboxylic acids were then derivatized with isobutyl chloroformate. After derivatization, the samples were mixed with hexane, and the hexane layer was collected for GC‐MS analysis. The quantitation of acetic acid, butyric acid, isovaleric acid, lactic acid, propionic acid, and succinic acid was performed using isotope dilution GC‐MS/MS. The absolute quantity of each SCFA was determined using a calibration curve measured for each analyte. For the quantification of amino acids in liver, tissues were extracted in 80% methanol (v/v) in water, processed, and measured using Thermo TSQ Quantiva Triple Quadrupole Mass Spectrometer with Vanquish MD UHPLC. All measurements were performed in the Proteomics & Metabolomics Core at Lerner Research Institute, Cleveland Clinic.

# **Supporting Fig. 1**


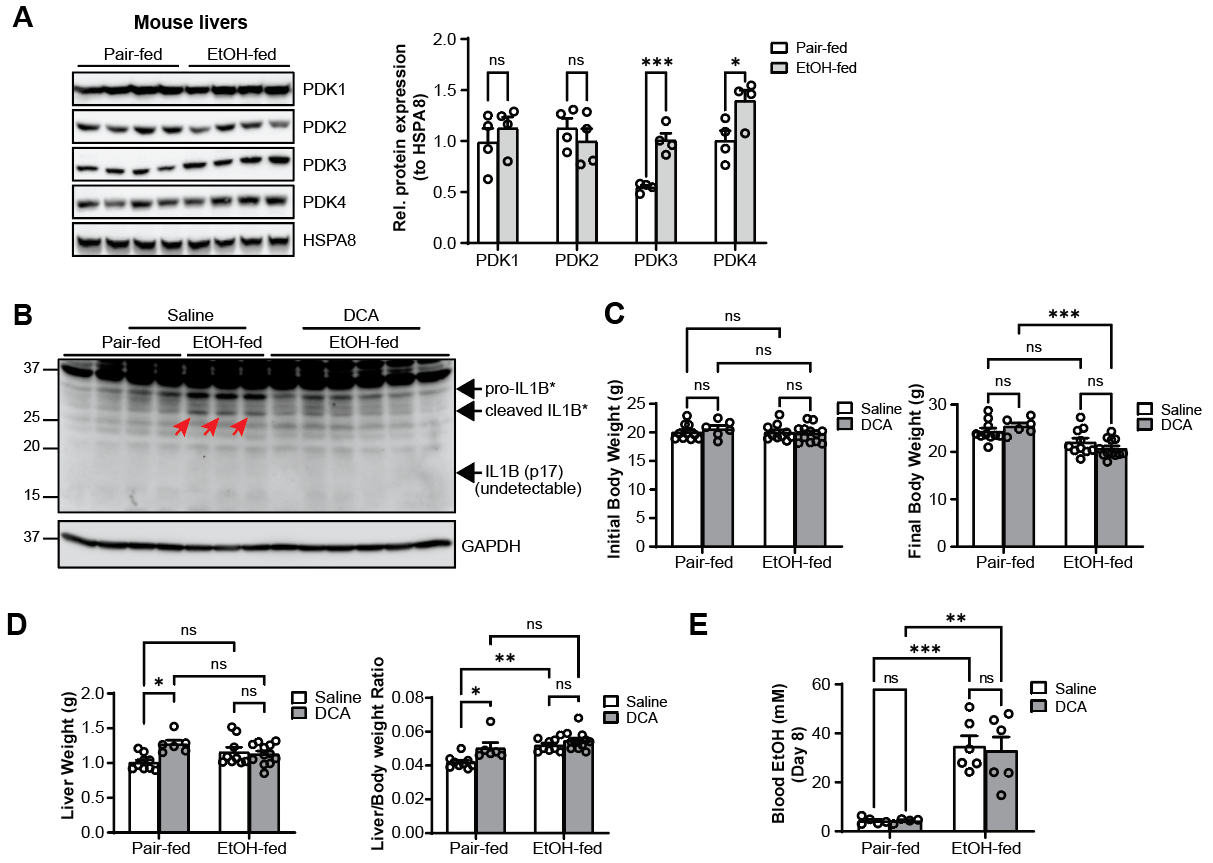


**Fig. S1A:** Representative Western blot (left) and quantification using ImageJ (right) of hepatic expression of PDK proteins in the mice exposed to the acute-on-chronic (Gao-binge) murine model of ALD. Data are shown as mean ± SEM, n = 4; **P* < 0.05, ****P* < 0.001; ns, not significant; by unpaired *t*-test.

**Fig. S1B:** Representative Western blot of IL1B protein levels in liver lysates from mice injected with saline or DCA in the acute-on-chronic (Gao-binge) murine model of ALD. The star (*) indicates that the bands for pro-IL1B and cleaved IL1B possibly overlay with non-specific bands. **Fig. S1C & S1D:** Comparison of initial body weight, final body weight, final liver weight, and liver/body weight ratio between different groups of mice in the acute-on-chronic (Gao-binge) murine model of ALD.

**Fig. S1E:** Blood ethanol concentrations of mice on day 8 in the acute-on-chronic (Gao-binge) murine model of ALD.

S1C, S1D, and S1E: Data are shown as mean ± SEM (n = 6 - 11 mice/group for S1C and S1D, pooled from two independent mouse feeding trials; n = 4 - 6 mice/group for S1E, from one feeding trial). **P* < 0.05, ***P* < 0.01, ****P* < 0.001, and *****P* < 0.0001 by 2-way ANOVA.

# **Supporting Fig. 2**

**Fig. S2:** Western blot analysis of IL1B levels in IL1B release assays. Bone-marrow-derived macrophages (BMDMs) from wild-type (WT) and *Pdk4*^-/-^ mice were primed with LPS (300 ng/ml, 3 h) and activated with ATP (1 mM, 1 h). IL1B levels in the cell culture medium (extracellular) and within cells (intracellular) were analyzed. Data are representative of 3 independent experiments.

# **Supporting Fig. 3**


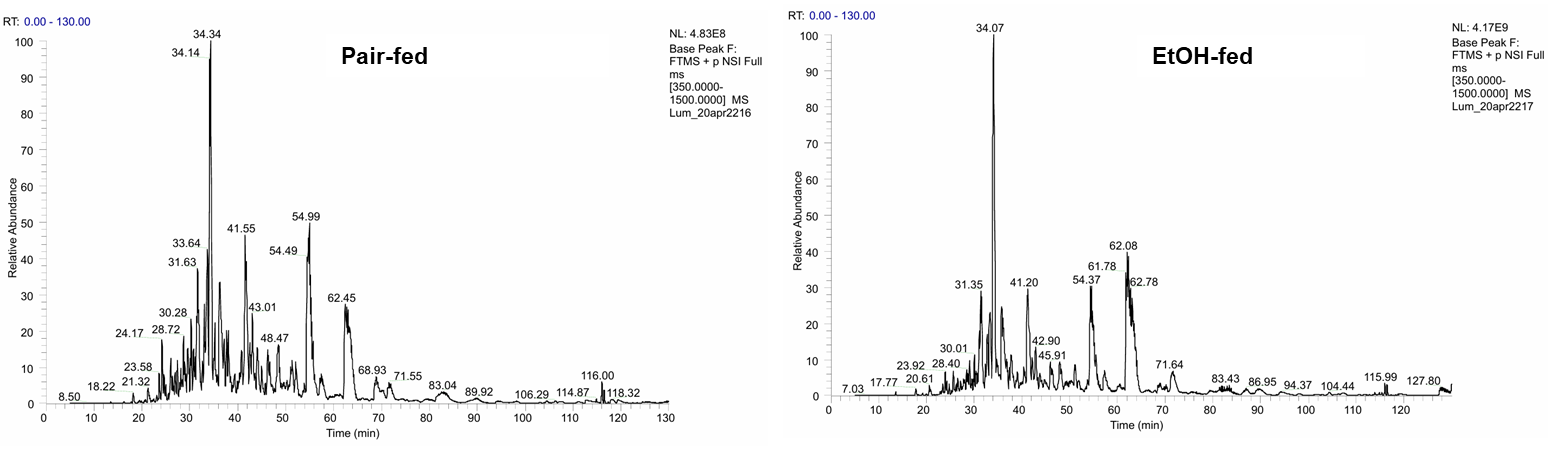


**Fig. S3:** Representative LC-MS chromatograms of the liver tissue proteome profiles in pair-fed (left) and EtOH-fed (right) mice in the acute-on-chronic (Gao-binge) murine model of ALD. Vertical axis, relative abundance; horizontal axis, retention time.

# **Supporting Fig. 4**

**Fig. S4A:** Correlation matrix of hepatic levels of amino acids in pair-fed (n = 4), ethanol-fed (n = 3), and ethanol-fed + EtOH (n = 6) mice in the acute-on-chronic murine model of ALD.

**Fig. S4B:** Hepatic concentrations of indicated amino acids in the groups of mice described in Fig. S4A. ns, not significant; analyzed by one-way ANOVA.

**Supporting references**

1. Wu X, Fan X, McMullen MR, et al. Macrophage-derived MLKL in alcohol-associated liver disease: Regulation of phagocytosis. *Hepatology.* 2023;77(3):902-919.

2. Wu J, Zhao Y, Park YK, et al. Loss of PDK4 switches the hepatic NF-kappaB/TNF pathway from pro-survival to pro-apoptosis. *Hepatology.* 2018;68(3):1111-1124.
